# The role of antioxidant micronutrients in the rate of recovery of burn patients: a systematic review

**DOI:** 10.1186/s41038-016-0044-x

**Published:** 2016-08-03

**Authors:** Mary Adjepong, Pius Agbenorku, Patricia Brown, Ibok Oduro

**Affiliations:** 1 Department of Biochemistry, College of Science, Kwame Nkrumah University of Science and Technology, Kumasi, Ghana; 2 Department of Surgery, Reconstructive Plastic Surgery & Burns Unit, Komfo Anokye Teaching Hospital, School of Medical Sciences, College of Health Sciences, Kwame Nkrumah University of Science and Technology, Kumasi, Ghana

**Keywords:** Burn, Micronutrients, Recovery, Infection, Tocopherol, Carotenoids, Ascorbic acid

## Abstract

Burn injury can be detrimental to the health of individuals, meanwhile victims lose proteins and micronutrients in wound exudates. Victims also experience extensive protein catabolism. These make them prone to malnutrition. Burn patients also suffer a lot of emotional trauma that reduce nutrient intake. The aim of this paper was to review primary evidence on the effect of antioxidant micronutrients on the recovery rate of burn patients. Electronic databases such as PubMed, BioMed, and Cochrane were systematically searched between January 1, 2014, and January 30, 2014. Keywords include vitamin A, vitamin C, vitamin E, ascorbic acid, zinc, copper, selenium, tocopherol, carotenoids, dietary intake, supplementation, wound healing, infection, recovery rate, and burn patients. The systematic search was done to retrieve all published data from 1990 to 2013. A total of 518 journal articles were obtained, and after the removal of duplicates, reviews, commentaries, and studies with non-human subjects, 11 papers were accepted for review. The review considered only papers that were published, and there might be some unpublished data that may have been omitted. Generally, the wound healing time and infection rates were reduced by the administration of the antioxidant micronutrients. The review revealed that there was no such published work in developing countries and children were excluded from most studies. It was also stated clearly that there was no uniformity in burn management; hence, there is a need for more studies on burn management in various populations.

## Background

Burn injury, which is the most devastating of all injury and a serious global public health crisis, is defined as an injury to the skin or other organic tissues primarily caused by heat or due to radiation, electric current, friction, or exposure to chemicals [1]. It is therefore classified under three main headings depending on the cause of injury: chemical burns are caused by exposure to acids or alkali; thermal burns are caused by hot water, hot oil, and open flame; and electrical burns are caused as a result of exposure to high voltage current or lightning. It can be fatal because it can lead to infection and death in some of its victims. It has been reported by World Health Organization that 265,000 people die globally from open fires alone and there are more deaths caused by scalds and electrical burns [1].

Infection, delayed wound healing, and extensive protein catabolism are among the main causes of mortality in burns. There is also a compromised immune system in burn patients. The main cause of immune suppression in burn patients is the presence of reactive oxygen species leading to delayed wound healing, and this increases the patient’s susceptibility to infection [2].

Medical nutrition therapy (MNT) remains pivotal in the management of burn injury. The goal of MNT is to maintain body mass, prevent starvation and specific nutrient deficiencies, improve wound healing, manage infection, and restore protein losses [3]. These goals are met through adequate portions of macronutrients and micronutrients.

Numerous studies have been done to examine the efficacy of different micronutrients on thermally injured patients [4]. Several reviews and commentaries as well as journal publications exist, but it is important to note that despite the numerous studies and journal publications in this area, little published data exist for antioxidant micronutrients and there are no published work found for Africa. Various electronic databases such as PubMed, BioMed, and Cochrane Library were searched using appropriate keywords. Some of the keywords include recovery rate of burn patients, micronutrient and burn patients, dietary intake, supplementation, vitamin A, vitamin C, vitamin E, ascorbic acid, tocopherol, carotenoids, copper, zinc, infection, sepsis, wound healing, protein catabolism, and protein turnover. All published data relevant to the scope of the study were included in the review.

The hypothesis of this review was that intake of antioxidant micronturients or its administration in burn patients improves recovery outcomes. The exposure measures were the intake of antioxidant micronutrients, and the main outcome measures were wound healing and infection.

## Review

### Search strategy

A systematic search was done, and all published data from 1990 to 2013 on the effects of various antioxidant micronutrients on the recovery rate of burn patients of all ages were retrieved. Papers with patients having varying degrees of burns suffered were included as well as papers with patients of both sexes.

The main search engines used include PubMed, BioMed, and Cochrane Library. The searches were conducted systematically to find the effect of each of the micronutrients on the various outcomes: wound healing time, infection rate, length of hospital stay, protein turnover, and catabolism.

Exposures for this review are vitamin A, vitamin E, vitamin C, zinc, copper, and selenium, while the main outcome was recovery rate, wound healing time, protein turnover, sepsis, infection, mortality rate and protein catabolism. The category of subjects included all age groups. Though most of the studies had age restriction, there were no exclusions to the ages of the patients in the search. The only works that were excluded were those that were done with non-human subjects.

**Fig. 1:**
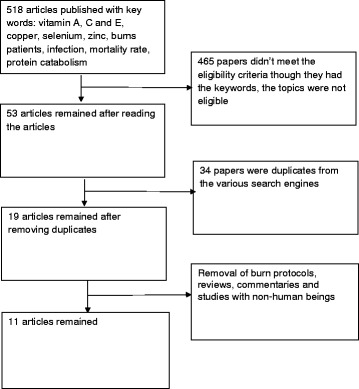
Literature searching and selection

Systematic literature searching and selection are presented in Fig. 1.

### Inclusion and exclusion criteria

Below were the inclusion criteria:

- Population: human population of all ages.
- Study designs: experimental and observational studies.
- Outcomes: outcomes measured include rate of recovery, length of hospital stay, infection, sepsis, wound healing time, protein turnover, protein catabolism, and mortality rate.

The following search results were excluded:

- Reviews.
- Reports on various protocols used for managing burn patients were excluded.
- Studies that were done with non-human subjects.
- Various case studies that did not meet the inclusion criteria.

### Search results

A total number of 518 journal articles were obtained from the various search engines. When the repeated articles were removed, articles that did not meet the criteria such as reviews and burn protocols were also excluded. At the end of the systematic search, only 11 remained.

### The findings on the studies involving effect of antioxidant micronutrient on the rate of recovery

Based on the results obtained from the various searches, a summary of the results were done and presented in Table 1.

**Table 1:** Summary of the main findings of the research

Author and country	Study designs	Aim of the study	Micronutrients involved and concentrations	Severity of burns (TBSA)	Age of patients	Outcomes	Gaps
Berger et al., Switzerland [6]	Randomized controlled trials (clinical trials) (*n* = 21)	To investigate the effect of large intravenous doses of trace element supplements on circulatory, antioxidant status, and the clinical outcomes after major burns	a) Copper (3.75 mg in IV and 2.7 mg in feeds) b) Zinc (37.5 mg in IV and 21.5 mg in feeds) c) Selenium (375 μg in IV and 90 mg in feeds)	Greater or equal to 10 %: greater than 20 %	16 to 65 years	• Higher circulation of plasma and skin tissue contents of selenium and zinc • Decreased pulmonary infection • Better wound healing	• Multicenter studies should be done
Berger et al., Switzerland [5]	Randomized controlled trials (clinical trials) (*n* = 21)	To assess the effects of trace element supplements on systemic substrate turnover and local protein metabolism during wound healing after major burns	a) Copper (59 μmol) b) Selenium (4.8 μmol) c) Zinc (574 μmol)	Greater or equal to 10 %: greater than 20 %	16 to 65 years	• Increase skin tissue concentration of selenium and zinc • Decrease protein catabolism	• Multicenter studies should be done
Berger et al., Switzerland [4]	Randomized controlled trials (clinical trials) (*n* = 41)	To determine the effect of trace element supplementation on nosocomial or intensive care unit-acquired pneumonia	a) Copper (2.5 to 3.1 mg/day) b) Zinc (26.2 to 31.4 mg/day) c) Selenium (315 to 380 μg/day)	Greater than 20 %	16 to 65 years	• Reduced pulmonary infections • Normalized plasma GSHPx activity, increased tissue selenium and zinc concentrations • An improved wound healing • A reduction in length of stay	• Dose response study should be done
Berger et al., Switzerland [9]	Randomized controlled trials (clinical trials) (*n* = 20)	To determine the effects of trace element supplementation after burn injury	a) 40.4 μmol of copper b) 406 μmol of zinc c) 2.9 μmol of selenium	Greater than 30 %	21 to 60 years	• Decrease broncho-pneumonial infections • Decrease hospital stay	• A better understanding of the effects of trace elements on neutrophil function and the acute phase response should be investigated
Al-Kaisy et al., Iraq [8]	Randomized controlled trials (clinical trials) (*n* = 58)	To determine the effect of zinc on recovery rate of burn patients	a) 15 mg of zinc	15 to 70 %	6 to 67 years	• Increase in antioxidant status as evidence by increase in GSH concentration • Decrease healing time	• None stated
Sahib et al., Iraq [7]	Randomized controlled trials (clinical trials) (*n* = 180)	To know the effects of various antioxidants on the recovery of burn patients	a) 400 mg of vitamin E and 500 mg of vitamin C/day b) 75 mg of zinc sulfate/day c) 100 mg of allopurinol/day d) 3 mg of melatonin/day e) 500 mg of *N*-acetylcysteine/day	15 to 40 %	Not stated	• Reduced incidence of infection • Reduced wound healing time and decrease in mortality rate	• None stated
Al-Jawad et al., Iraq [2]	Randomized controlled trials (clinical trials) (*n* = 60)	To explore the variable effects of *N*-acetylcysteine on wound healing in burn patients	*N*-acetylcysteine (500 mg/day)	15 to 40 %	20 to 40 years	• Decrease time of hospital stay • Decreased healing time	• None stated
Calds-Courtis et al., Canada [14]	Prospective cross-sectional (*n* = 23)	To know the effect of vitamins C and E and zinc on oxidative stress on burn patients	a) Ascorbic acid (1000 mg/day) b) Zinc (50 mg/day)	10 to 93 %	17 to 80 years	• Zinc concentration returned to normal values after 3 weeks • Zinc supplementation did not have adverse effect on serum copper concentration • Supplementation did not lead to gastrointestinal side effects	• None stated
Barbosa et al., Brazil [15]	Randomized controlled trials (clinical trials) (*n* = 32)	To know the duration of zinc supplementation and the effects it has on gastrointestinal side effects	a) Vitamin C (600 to 2700 mg) b) Vitamin E (270 to 1080 mg) c) Zinc (6 to 22 mg)	Greater than 10 %	2 to 15 years	• Decrease in wound healing time	• Dose response studies should be done
Rock et al., USA [16]	Prospective randomized trials (*n* = 27)	To know the effect of the intake of carotenoids	a) Beta carotene (30 mg/day)	Greater than 20 %	18 to 65 years	• Increase in plasma concentration of beta carotene	• A study to know the ability of antioxidant micronutrients to influence risk of secondary tissue injury and disease should be explored
Zhang et al. [10]	Randomized controlled trials (*n* = 35)	To know the effect of vitamin E on lipid peroxidates	Vitamin E (100 mg/day)	Severe burns: greater than 10 %	14 to 62 years	• Concentrations of vitamin E increased while lipid peroxides decreased	• Antioxidant therapy in burn patients should be explored

*TBSA* total body surface area; *GSH* glutathione

#### Study designs

The prevailing study designs used in the various studies were clinical trials where the micronutrients were administered to the patients intravenously, orally, or through enteral feeding and various outcomes were measured after the intervention.

#### Study population, setting, and country

The population of the various studies were similar. The subjects were healthy males and females and children and adults mostly between the age of 6 and 60 years. The exclusion criteria were mainly patients who suffered from other chronic diseases such as renal complications in addition to the burn injury. The percent of total body surface area (TBSA) burned of the patients in all these studies ranged from 10 to 93 %.

Most of the studies reviewed in this paper were done in Switzerland [4–6]. There were studies in Iraq [7, 8] and there was a study each in USA, Canada, and Brazil. There was no study done in Africa.

#### Antioxidant micronutrients considered in the study

The antioxidant micronutrients considered are vitamins A, C, and E, copper, zinc, and selenium. In one study, the efficacy of various antioxidants was compared among various groups with respect to the % TBSA burned [7].

Apart from the micronutrients that were used, *N*-acetylcysteine was the most efficient antioxidant used [2, 7].

#### Main findings

One remarkable measure of the rate of recovery is the reduced wound healing time as reported by most of the studies [2, 5, 7]. Apart from that, there was a decrease in mortality rate that was evidenced by shortening in hospitalization period and reduced incidence of infection [2, 7]. In addition, decreases in protein catabolism as well as increases in the concentration of trace elements in serum and skin tissues of the patients after the clinical interventions were also discovered [5].

The outcome from the studies as reported by Berger et al. revealed that the administration of trace elements caused a reduction in nosocomial infections such as broncho-pneumonial infections [4, 9]. It was also reported that the micronutrient levels were low at the initial stages of the burn injury. However, there was an increase in serum concentration of antioxidant nutrients within a few days of administration of the antioxidant minerals [4–9] as well as a decrease in serum lipid peroxides [10].

It should also be noted that 5.58 μg/ml was the average vitamin E level circulating at the initial stages of the burn injury, but after 20 days of administration of vitamin E, the level increased to 8.58 μg/ml [10]. Serum glutathione (GSH) also increased from 2.2 Mu/mg protein at the initial stages of burns to 16.8 Mu/mg protein at day 20. This gives an indication of increase in circulating antioxidants after administration of nutrients.

Moreover, a normalized antioxidant status of patients who received the interventions as evidenced by an increase in GSH concentration [2] in the plasma is a good indication of the positive effect of trace element supplementation on burn patients.

The efficacy of antioxidant micronutrients on the recovery rate of burn patients can hence be ascertained with these positive results and indications.

### Research gaps

According to the results, it is important to note that no published work exists for Africa and this creates a research gap. African women and children are more vulnerable to or are at high risks to burn injury, as a result of their lifestyle and their role in preparing the household meals. In Africa, poverty, illiteracy, and urban migration lead to overcrowding and unemployment. In most cases, mothers hunt for jobs and leave their children unattended, exposing these children to the risk of burn injury. In addition, the use of open flames in cooking is a serious risk factor.

It was also evident from the review that there was little attention on children below 6 years of age (only one study included them), and this gives rise to a research bias. There are several children in Africa who are malnourished; hence, a study to know how they are affected by burn injury will be a worthwhile study. It has become important because, in the various developmental milestones, children need nutrients for growth and development; hence, a study to know what happens to them under burn injury will be an interesting one.

All the studies reviewed gave promising results about the safety and efficacy of antioxidant nutrients on burn patients, but they all had one thing in common: small sample size. It has therefore been recommended that large multicenter trials are required to confirm these encouraging data as well as explore its applicability in burns and critical illness [5, 6]. Another study also recommended that, since trace elements have a dose response effect, there should be a study to find the appropriate doses and duration of treatment in burns for utmost recovery [5].

Currently, only 11 papers in this field have been published in 23 years. They varied in the parameters reported: immune cell functions, antioxidant levels, and outcome of the wound healing process. In general, these reports indicate that antioxidant supplements benefit burn patients, but more studies with careful clinical designs are required to find out the variety of the antioxidants, the dose, the timing, and the duration of the antioxidants to be used and the specially targeted parameters. These specific trials can include “the pulmonary function in burn patients with inhalation injury.” Extensive non-human studies have been reported, but it is also important in translating their results into clinical practice.

Neutrophils provide the first line of defense against infection by its phagocytic action on microorganisms and debris. Though some studies reported the potential role of trace elements in increasing the level of phagocytes, it has been recommended that a better understanding of the effects of trace elements on neutrophil function and the acute phase response should be investigated.

The role of antioxidant micronutrients cannot be overemphasized in these studies; hence, a study on antioxidant therapy in burn patients should be seriously considered [10].

## Discussion

The various results obtained from the studies indicate that the effect of multiple antioxidants on the rate of recovery of burn patients is outstanding. In the study by Berger et al., it is noted that the nutrients used as an intervention had a positive effect on wound healing, infection rate, protein turnover, and protein catabolism [4]. Studies by Al-Jawad et al. and Al-Kaisy et al., where single antioxidant nutrients were used, showed that there was an improvement in only one outcome variable [2, 8].

The synergistic role of the various micronutrients in the recovery outcomes was evident; hence, the role of individual micronutrients in the recovery outcomes can be challenging to know. Hence, a study to find out the role of each of the micronutrients will be beneficial, as Berger et al. stated that selenium is a major nutrient that can be investigated alone because of the role it plays in oxidative stress as seen in animal models [4].

The effect of micronutrients and the role they play in immunity cannot be overemphasized. Some studies that measure infection rate have established that trace elements may have an effect on the neutrophil level in an individual. It is reported by Berger et al. [4, 9] that trace element supplementation reduced infection rates. Ross et al. report that selenium is likely to play a role in immune function and hence is a major research area [11].

Zimmerman et al. and Winichagoon et al. reported that supplementation of micronutrients such as zinc in young people can help in their growth and development and cognitive function [12, 13]. Zinc plays crucial roles in oxidative stress, bone formation, taste acuity, and the stabilization of membranes and also in the formation of connective tissues [11]. A burn child is likely to be deficient in zinc and becomes vulnerable to all kinds of infection due to prolonged deficiency after burn injury.

Most of the antioxidant micronutrients were given in doses higher than their recommended daily allowances; it was therefore considered as a therapeutic dose. As to whether the required quantities of the micronutrients can be obtained in the diets of the patient remains unknown. Interventional studies dominated the results of this study. There was no study done on a cross-sectional prospective basis; hence, designing a study in that manner can give detailed information about the situation in developing countries.

## Conclusions

Based on the forgoing review, the use of antioxidant nutrients such as vitamins A, C, and E, copper, zinc, and selenium confers health benefits to burn patients. This ranged from a reduction of infection rates, reduction in wound healing time, and shortening of the length of stay in hospitals. It is evident from this review that there are several gaps with respect to nutritional management. There is also a need for more work to be done in the African population where the inhabitants are more prone to burn injury. This paper therefore urges scientists to take an active role in burn research to help the vulnerable people in society.
